# Exploring Ukrainian Refugee Women’s Beliefs and Concerns About Healthcare Systems, with a Focus on HPV Immunization Practices: A Mixed-Methods Study on Forcibly Displaced Populations in Romania

**DOI:** 10.3390/healthcare13141744

**Published:** 2025-07-18

**Authors:** Teodora Achimaș-Cadariu, Andrei Pașca, Delia Nicoară, Dan Lucian Dumitrașcu

**Affiliations:** 1 Faculty of Medicine, “Iuliu Hațieganu” University of Medicine and Pharmacy, 400012 Cluj-Napoca, Romania; 2 “Prof. Dr. Ion Chiricuta” Institute of Oncology, 400015 Cluj-Napoca, Romania

**Keywords:** vaccine, HPV vaccine, vulnerable population

## Abstract

**Objectives**: Scarce data are available regarding preventive medicine in forcibly displaced populations especially regarding non-communicable diseases like neoplasia, while even more limited data are available on Ukrainian refugees in Romania. To address this research gap, the present analysis was performed to investigate Ukrainian refugee women’s beliefs, attitudes, and opinions towards the Romanian and Ukrainian healthcare system in a comparison model while focusing on the HPV immunization rates and factors influencing the uptake for themselves and their children. **Methods**: Participants were recruited using the snowball sampling method through their General Practitioner (GP) and a health mediator. **Results**: In total, 105 women completed the online or physical survey. The mean age was 50 years. In total, 40% of women had not been to a gynecological check-up in 3 or more years, and more than 56% had never been screened. Only four were vaccinated against HPV, and none remembered which type of vaccine was dispensed or how many doses were utilized. The primary hindrances to accessing health services or immunization programs were language barriers, financial burdens, and a lack of information. Respondents’ general distrust of health systems and healthcare workforces were recurrent themes. Relationship status, living arrangements, and previous engagement in screening practices influenced immunization rates. Perceiving the healthcare officials as proactive concerning optional vaccination programs such as HPV immunization and actively receiving recommendations drove respondents to pursue vaccination. **Conclusions**: This analysis offers a foundational insight into the specific needs of refugee women. It can guide the development of effective public health interventions to improve health outcomes and vaccination rates among Ukrainian refugees in Romania. Tailored preventive campaigns with adequate native language information and prompts from medical experts in designated centers should be deployed to ensure inclusive tactics for vulnerable populations.

## 1. Introduction

Primary and secondary prevention proved to be efficacious pillars of a sustainable healthcare system while lessening the incidence and mortality attributed to miscellaneous communicable and non-communicable diseases, including cervical cancer (CC) [1]. This neoplasia is among the few oncological pathologies that could potentially be eradicated as a public health issue via prophylactic measures, such as HPV vaccination and cervical cancer screening (CCS), as per the World Health Organization’s (WHO’s) suggestion [2]. In a previous observational investigation, a reduction in CC rates as high as 87% was highlighted in the 20- to 30-year-old group when vaccinated against HPV at the age of 12–13 [3]. A modeling analysis for Low- and Middle-Income Countries (LMICs) demonstrated that scaling up screening to once or twice in a lifetime could avert up to 400,000 deaths amenable to CC, translating to a reduced mortality by more than 34% [4]. Until vaccinated cohorts reach adulthood, vaccination will likely not reduce mortality rates by itself, but combined with screening and early treatment, it was estimated that by 2070, such preventive methods could avert more than 13 million deaths [4].

Although these practices are widely available worldwide, significant discrepancies have been reported throughout regions regarding implementation and the addressability of the targeted populations [5]. Within European countries, gender-neutral programs, the availability of catch-up strategies, vaccination rates, and organized screening programs vary significantly [6]. Although more than 60% of countries proposed vaccination targets, only two European countries have reached the WHO proposed landmarks of immunization rates in 15-year-olds [6]. Adding to alarming statistics, a large study on CCS in 202 countries unveiled that only around 10% of women aged 30 to 49 from LMICs have ever been screened, as opposed to 83% in High-Income Countries (HICs) [7]. Furthermore, the COVID-19 pandemic has reduced vaccination rates and screening coverage over the past few years [8]. The aforementioned discrepancies emphasize the importance of the timely and wide-scale implementation of strategies alongside appropriately tailored programs to increase HPV immunization uptake. Previous investigations strived to uncover solutions by investigating gender-neutral programs [9] and dose-sparring options [9,10].

Since early 2022, the conflict in Ukraine has displaced 6.3 million refugees throughout European countries, and as of 2025, over 180,000 Ukrainian refugees were recorded on Romanian territory alone. Over 190,000 Ukrainians applied for asylum, temporary protection, or other forms of national protection schemes in Romania [11]. Significant efforts have been made to ensure that these massive population movements will be provided with appropriate health access. Public health considerations for controlling and preventing infectious diseases have been a focus of such actions [12], mainly to contain vaccine-preventable disease outbreaks. Considering the logistical implications of said efforts, non-communicable diseases such as oncological pathologies were deprioritized, and screening practices among displaced populations were less explored. It was previously uncovered that inadequate addressability to screening programs in refugee populations [13] and very low immunization coverage and completion rates [14] were noteworthy hindrances to public health outcomes. Lower uptake for HPV immunization was revealed among girls with a refugee background compared with native populations [15], with an Odds Ratio (OR) of 0.44. The trend continued for catch-up programs as well, uncovering lower rates for refugees with an OR of 0.61 [15]. Income, country of origin, and length of residence were characteristics influencing the immunization uptake [15]. Although very little data were available on the Ukrainian refugees’ immunization and screening practices, cultural, financial, and linguistic issues were deemed as outstanding barriers in accessing the healthcare system in Romania [16].

In Romania, the HPV immunization campaign started in 2008, and a CCS program was implemented in 2012 [17,18]. Despite these preventive measures being readily available for more than 10 years, Romania ranks first in Europe in terms of incidence and mortality attributable to CC [19,20]. In Ukraine, 4756 new CC cases are diagnosed annually, being the second most common cancer in women aged 15–44 years old [21]. CCS has been available since 2014. In 2018, the National Action Plan for Non-Communicable Diseases was implemented because 80% of deaths were caused by non-communicable diseases, including oncological ones [22]. As of today, no HPV immunization program has been implemented, but the Ministry of Health of Ukraine plans to introduce the HPV vaccination into the National Immunization Schedule, starting from 2026, addressing girls aged 12–13 years old [23].

The non-communicable disease challenge posed by the considerable influx of refugees and asylum seekers in Romania due to the current conflict over the border should be evaluated accordingly. Resolutions must be deployed in time, with preventive care as one of the most important pillars in mind for a public health system integrating such populations. Vaccine hesitancy among immigrants and refugees was formerly reported [24], and regardless of the country of origin, most refugees will not complete their vaccination schemes [14]. In Romania, more than 200,000 Ukrainians have been granted temporary protection [25]. Consequently, particular awareness and dedicated endeavors must be undertaken to ensure inclusive tactics are used to reach out to these underserved populations, specifically when stemming from areas with already subpar coverage [26]. This is especially important since Romania is a nascent state of developing structured screening programs and immunization campaigns. Therefore, equitable strategies must be deployed as early as possible to ensure favorable public health outcomes.

The present analysis strived to fill this research gap by investigating Ukrainian refugee women’s beliefs, attitudes, and opinions towards the Romanian and Ukrainian healthcare systems in a comparison model while focusing on the HPV immunization rates and factors influencing the uptake for themselves and for their children. It also used a mixed-methods approach by performing both a quantitative analysis on demographic data and variables influencing vaccination willingness and rates while also refining the results with a qualitative synthesis that dives deeper into the hindrances experienced by this population in accessing the Romanian healthcare system.

## 2. Materials and Methods

### 2.1. Participants

The sample population consisted of Ukrainian women refugees aged 18 or older living in Cluj-Napoca, Romania. The respondents were recruited using the snowball sampling method through their General Practitioner (GP) (T.A-C.) and a health mediator and translator who disseminated the questionnaire and helped respondents complete the questionnaire when necessary. The snowball sampling method is a non-probability sampling method where existing study participants (in this case, refugees enrolled with their GP or in contact with the health mediator) recruit future subjects, who are potentially harder to reach. This was deployed to reach refugees who were not enrolled with a GP or in contact with health mediators, therefore striving to pool a more diverse population. The questionnaire was distributed both online and physically. Data were collected between March 2023 and March 2024.

### 2.2. Instruments

The questionnaire included an informed consent section and 57 questions that were conceived to assess the existing literature and to collect demographic data. Close- and open-ended questions were used to uncover the barriers and facilitators women could face regarding the healthcare system and immunization process. The questionnaire included two sections: demographic characteristics (including history of gynecological check-ups, CCS, and vaccination status or previous refusals and HPV vaccination), while the second part studied the interaction with the Romanian and Ukrainian healthcare systems. The questionnaire can be found in Supplementary Materials S1. It was conceived of in English (as shown) but was translated to be applied in Ukrainian with the help of a native speaker and authorized translator.

### 2.3. Data Analysis

The online results were exported into an Excel file together with the physical questionnaire. All data were translated by an authorized translator who was also a native speaker of the Ukrainian language. Data analysis was undertaken using JASP Statistics software, v 0.19.3 [27].

For the quantitative investigations, Odds Ratio (OR) was deployed to assess the correlation between dichotomous variables, with a Confidence Interval (CI) = 95% and a p-value of 0.05 [28]. For the power analysis, OR was considered an indicator for the effect size, with the following values, 1.5, 2.5, 4, and 10, showing small, medium, large, and substantial effect sizes [29]. The Chi^2^ test was used to assess statistical correlation for other non-dichotomous variables. When the expected theoretical frequencies for subgroups were less than 5 in more than 80% of cases, or less than 1, variables were grouped. If grouping was no longer feasible, a continuity correction was used for dichotomous data, or Fisher’s exact test was deployed for other variables. For the Chi^2^ test, Cramer’s V was used as an indicator for the power analysis, considering the degrees of freedom for each variable [30]. For continuous variables, Shapiro–Wilk’s test was used to assess the normality of distribution. Mann–Whitney or Student’s Test were used for the independent samples *t*-test, depending on the normality of distribution.

The qualitative analysis used open-ended questions to explore participants’ experiences with healthcare systems and immunization schemes. Content thematic analysis, a qualitative method used for data analysis, was deployed. Content thematic analysis involves systematically coding and merging data into themes while quantifying recurrent themes [31]. An inductive approach was used to identify emerging patterns and themes [32]. Thus, this study was categorized as mixed methods, as it used both quantitative and qualitative methods for assessing the desired variables.

The study was conducted with ethical approval from the Prof. Dr. Ion Chiricuta Institute of Oncology, Cluj-Napoca, Romania, number 256/2 February 2023.

## 3. Results

### 3.1. Descriptive Statistics

In total, 105 women completed the survey, of which six questionnaires were completed online. The mean age of the participants was 50 years; over 55% of respondents had a high education level (ISCED 5 and above), with mean values of just over 5. On average, participants settled in Romania for one year prior to completing the survey, while more than half (51.42%) had not traveled back to Ukraine since. Over 33% only return once or twice yearly (see Table 1).

Concerning family structure, most of them resided in Ukraine with their children and extended family (husband, mother, nephew). However, their living arrangements in Romania were more restricted, most living with their family or husband, while almost 38% lived alone, with relatives or acquaintances. In total, 80% of women were married or in a relationship, while more than 90% were religious. Most were unemployed (almost 64%) in Romania, while the unemployment rate in Ukraine was sub-50%. A little over 91% of them had self-assessed poor and very poor proficiency in the Romanian language. Table 2 depicts specific data.

Table 3 spotlights the gynecological antecedents; 40% of women had not been to a gynecological check-up in 3 or more years, while more than 56% never had a Babes–Papanicolaou smear or an HPV test. Of the respondents who did perform one of the procedures, only 2 reported abnormalities, and 59 did not answer the question. In total, 13% of women claimed to be unvaccinated, while only 10% recalled refusals. Regarding the quantum of respondents who received a vaccination recommendation, more than 64 did, while only 41% recalled an HPV vaccination recommendation; of those who did, almost 40% received the recommendation from their Romanian family doctor and almost 27% from other doctors, but still in Romania.

Only four (3.81%) women were vaccinated against HPV, and none recalled which type of vaccine was administered or how many doses. Of the unvaccinated, 25 (24.75%) would have liked to get vaccinated.

Most participants indicated they had access to primary prevention services in Ukraine (n = 67), whereas more than half reported lacking similar access in Romania (n = 58). Most women reported undergoing all the mandatory vaccination schemes and that they were vaccinated against COVID-19. Many received recommendations (n = 59) from healthcare professionals (HCPs), including family doctors and pediatricians, with a few explicitly noting Ukrainian doctors. When asked about the types of vaccinations recommended, most mentioned COVID-19, followed by flu shots, while only one mentioned receiving a recommendation for tetanus or HPV. A small number of women (n = 11) refused certain vaccines, citing reasons such as a need for more reliable vaccines, pain and discomfort, and being elderly or having cancer. Additionally, 98 respondents did not answer the question regarding vaccine refusals.

Regarding the children’s vaccination program, most parents said their children received all the mandatory vaccines. Generally, parents did not refuse any vaccinations, except when their child had the flu or experienced an adverse reaction to a previous vaccine, in which case the child did not receive the second dose.

Most participants (n = 52) were unaware of the differences between the vaccination schedules in Romania and Ukraine, and only 14 had experience with the vaccination system by vaccinating their children. Few mentioned that the vaccination program is the same, while others thought it is better than Ukraine’s.

### 3.2. Quantitative Analysis

#### 3.2.1. HPV Immunization Status

Regarding demographic data, the relationship status of the participants, living arrangements in Romania, and previous engagement in screening procedures were correlated with the vaccination status. The single/divorced/widowed group were more likely to be vaccinated against HPV than their married or in a relationship counterpart. Respondents living alone had higher chances of being vaccinated while women who had previously engaged in screening procedures (either a Pap smear or an HPV test) were more likely to be vaccinated. All analyses required a Fisher’s exact test for further clarification, but the statistics could not be calculated due to zero events in some groups. Education levels, religion, living arrangements in Ukraine, income or employment, language levels, age, or length of residence in Romania did not seem to influence the vaccination rates. Results can be found in Table 4.

Regarding previous experiences with the healthcare system, women refugees receiving non-medical recommendations for other vaccines or for HPV vaccination were more likely to be immunized against HPV. Interestingly, a previous vaccination refusal correlated with the vaccination status. The first analysis also required a Fisher’s exact test that could not be calculated due to the observed events being zero for a subgroup (no women previously receiving a doctor’s recommendation for any vaccine were immunized against HPV). The last visit to a gynecologist or having previously received an abnormal screening result did not correlate with the vaccinal status of the respondents. Table 4 shows the exact results.

Statistics regarding women’s beliefs and concerns about the healthcare system highlighted certain profiles of respondents with higher chances of being vaccinated:

− Ukrainian refugees **believing that sufficient information is available in the Ukrainian language** about the HPV vaccination programs in Romania;
− Those who **perceived Ukrainian authorities as proactive about HPV** vaccination were more likely to be vaccinated;
− Those who **believed there are enough vaccines for everyone in need in Ukraine**;
− Respondents who were **aware of slightly different vaccination schedules** in Romania and Ukraine;
− Women who had **previously experienced the Romanian vaccination system**.

#### 3.2.2. Willingness to Vaccinate Against HPV

Of the unvaccinated population, certain demographic characteristics seemed to influence the vaccination uptake: living arrangements in Romania and the desire to vaccinate their children against HPV. Other variables did not seem to influence participants’ willingness to engage in HPV vaccination.

Regarding the intricate relationship with the medical services, respondents who frequently used medical or dental services when traveling back to Ukraine or those believing that more native language information could increase uptake were more likely to want to be vaccinated against HPV than their counterparts. Women perceiving the Ukrainian authorities as encouraging and those perceiving Romanian authorities as better than Ukrainian ones were also more likely to get vaccinated. Preferences for state or private hospitals or access to primary prevention did not alter respondents’ willingness to vaccinate against HPV. Data can be found in Table 5.

#### 3.2.3. Vaccinating Children Against HPV

Statistically meaningful distinctions were observed regarding women’s preference to vaccinate their children against HPV with respect to their income and employment rates in Ukraine and number of children. Educational levels, relationship status, religion, living arrangements in Ukraine or Romania, language proficiency, age, length of residency in Romania, or frequency of visits to Ukraine did not seem to influence the vaccination rates. Income and employment in Romania also did not hinder vaccination intent for children, unlike income and employment in Ukraine.

A significant number of women (n = 69) did not receive any recommendations for the HPV vaccine. Factors driving respondents to pursue HPV vaccination for their children can be found in Table 6. Additional variables such as the last visit to a gynecologist, ever engaging in screening, having previously received an abnormal Pap smear or HPV test, parents’ vaccination status, or previous child vaccination refusals did not influence women to pursue HPV immunization for their children.

### 3.3. Qualitative Analysis

#### 3.3.1. Access to Healthcare in Ukraine and Romania

In total, 90 women stated that their access to healthcare changed after moving to Romania, and this was mostly attributed to **healthcare professionals’ expertise and attitude** (n = 47). This highlighted a more patient-centered approach influenced primarily by doctors’ behavior, fostering a safe environment where patients felt genuinely respected and helped according to their needs.

“*Because doctors are better specialists and their attitude with patients is better.*”

(P11, 45 years old, moved to Romania 12 months ago)

**The contrasting healthcare systems** revealed that Romanian health services were perceived as taking better care of the individual while hospitals were more accessible, cleaner, and better equipped (n = 16).

“*The hospitals have all the equipment they need, clean hospitals, good food. If you have insurance, then the medication is free too.*”

(P83, 68 years old, moved to Romania 12 months ago)

Interestingly, closely related to the healthcare system differences, **the effectiveness of medication** was mentioned by women. Respondents believed they were more likely to receive comprehensive care and proper medication for their pathologies, highlighting the differences between the two countries and emphasizing certain medical practices in Ukraine, where patients may feel that medication does not always meet their needs:

“*In Ukraine, every disease is treated with perfusions, and it seems like it is treated differently.*”

(P92, 63 years old, moved to Romania 6 months ago)

Those differences were attributed to geo-political factors (n = 7):

“*You can feel it—Europe.*”

(P82, 26 years old, moved to Romania 15 months ago)

Some women (N = 5) humorously remarked on the similarities between the two healthcare systems, “*the fact that they are called doctors*” (P64, 44 years old, moved to Romania 6 months ago).

The **financial burden** was mentioned as a primary reason for a preference towards state healthcare services in Ukraine (n =77), highlighting the high costs associated with private healthcare and accentuating the disparity between the wealthy and the poor:

“*Poor people go to the state (hospitals), in private—rich ones*”

(P92, 63 years old, moved 6 months ago)

In Romania, there is a noticeable emphasis on **making healthcare accessible to all** and trying to alleviate some financial burdens associated with medical care. The situation in Ukraine was described as quite different. Access to treatment often hinges on one’s financial means; those who can afford it will receive care, while individuals who cannot have to remain at home without necessary medical attention.

“*Here (in Romania), I have the impression that it is more focused on people’s health. People can be treated for free. In Ukraine, sadly, if you have money, you can get treated; if not—you stay home.*”

(P87, 37 years old, moved to Romania 12 months ago)

Nevertheless, many respondents chose to use medical services in their home country. The most frequent medical services used by women who traveled back to Ukraine (n = 73) were dentistry (n = 31), cardiology (n = 9), and gynecology (n = 7), due to financial considerations (n = 7). Some women preferred Romanian medical services (n = 62), 36 preferred both, while only 7 preferred the Ukrainian ones. Nevertheless, preferences did not translate into practice.

This burden seems to be alleviated by the free services offered by **state and private hospitals** in Romania (n = 38).

“*In Romania, I got treated in state and private hospitals. Many times; you can find funds, where you can be treated for free*”

(P28, 75 years old, moved to Romania 22 months ago)

Additionally, a preference for public healthcare emerged when multiple consultations were necessary, as costs can add up; the healthcare professionals (HCPs) could help navigate the healthcare system.

“*I prefer the public sector because doctors and services in Romania are costly, and also because I have health problems now and I need to investigate the causes, which means more than one visit to the doctor, so I needed a consultation with a family doctor and referrals to highly specialized doctors to understand where to go*”

(P104, 24 years old, moved to Romania 5 months ago)

**Lack of language knowledge** was mentioned as a factor that could hinder the willingness to access healthcare services in Romania (n = 11). Difficulties accessing the healthcare system can accentuate the impediments already faced by women or those reluctant to seek medical care, modifying their willingness to seek help. One participant suggested the following:

“*The translation of instructions or documents in medical institutions should at least be available in English.*”

(P46, 33 years old, who moved to Romania 12 months ago)

Women mentioned **that doctors’ goodwill and positive attitude** encouraged them to seek medical care. Additionally, there is a desire for more **efficient processes** to reduce consultation waiting times and increase the number of emergency departments and hospitals to improve access to the Romanian healthcare system. A 63-year-old participant who moved to Romania six months ago said the following:

“*I am grateful to these doctors who know what they have to do. It would be better not to stay in immense waiting lines. Being sick is very hard.*”

(P92, 63 years old, moved 6 months ago)

#### 3.3.2. Immunization Beliefs and Practices

Thirty-nine women perceived the **attitude of** Ukrainian **authorities regarding vaccination** as positive. At the same time, eighteen stated a passive attitude, and nine attributed negative attitudes to the changes in the healthcare system due to the current conflict. However, there were polarized responses. Women perceived the government’s involvement as focusing on access and health for the population.

“*I think the government does everything possible to provide vaccines for the population*”

(37 years old, moved to Romania 15 months ago)

Some respondents remarked that a **lack of interest from the authorities** attributed to the vaccination being free, pointing out skepticism regarding the quality of the vaccines. They felt responsible for their health:

“*Since this is a free procedure, the Ukrainian authorities are not interested in it. If you want to do something at your own expense, you can do it in a private clinic, but you must wait a long time for free, and you cannot always be sure that the government has bought a good vaccine*”

(P104, 24 years old, moved to Romania 5 months ago)

“*This is everyone’s choice and responsibility. They do not care.*”

(P104, 24 years old, moved to Romania 5 months ago)

Ukrainian women perceived **Romanian authorities as having a more positive attitude regarding general vaccination** (n = 64), focusing on helping the community and granting access to the entire population. Interestingly, respondents perceived the vaccines as more effective:

*I do not know. I think here, authorities think a little bit of ordinary people*

(P25, 48 years old, moved to Romania 12 months ago)

Forty-two participants observed **differences in primary prevention between Romania and Ukraine**, specifically noting the **greater availability of vaccinations**, including those for Pneumococcus. One participant stated the following:

“*In Romania, they vaccinate against Pneumococcus, but in Ukraine, they do not*”

(P10, 32 years old, who moved to Romania one month ago)

**Ukrainian authorities** seemed to be **less encouraging towards HPV vaccination** (n = 24). Almost one-third of the participants received no information regarding the HPV vaccine in Ukraine (n = 33). In contrast, those who received the primary source of information were the healthcare professionals (n = 24).

Authority-related factors that could facilitate HPV vaccination in Ukraine were more information regarding the vaccine (n = 35), quality and effectiveness of the vaccine (n = 18), free access (n = 9), and mandatory (n = 3) vaccination.

Ukrainian women perceived Romanian authorities as having a more positive attitude regarding the HPV vaccine (n = 33) and believed that they would benefit from better access (n = 32). Eighty-nine women mentioned that there is a lack of information in the Ukrainian language in Romania. The primary sources of information regarding the HPV vaccine in Romania were from HCPs (n = 20), while 22 stated that they did not have any sources of information. Authority-related facilitators towards Ukrainian women’s intention to vaccinate in Romania would be information regarding the HPV vaccine (n = 50), with a preference for native language information stated by five women. Free vaccination was also noted (n = 83).

Women’s perspectives regarding HPV vaccination were commonly positive (n = 57). The main reasons for **HPV vaccination refusals** were insufficient information (n = 30), fear of side effects (n = 11), lack of trust (n = 9), and personal decision and responsibility (n = 7).

“*Not many people do know about this vaccine*”

(P52, 62 years old, moved to Romania 12 months ago)

Prevention, including health benefits, was the most cited reason for **HPV vaccination acceptance** (n = 26),

“*To prevent the spread of HPV and to protect women from CC*”

(P63, 66 years old, moved to Romania 7 months ago)

Fear was also a contributing factor in the intention to vaccinate, facilitated by the desire to be healthy, “*When people are afraid for their health*” (P31, 58 years old, moved to Romania 9 months ago) and at the same time by a fatalistic view “*this uncurable disease*” (P92, 63 years old, moved to Romania 6 months ago). Other reasons that could facilitate vaccination intentions were more information (n = 18), the quality of the vaccine (n = 6), and the mandatory character of immunization (n = 2).

## 4. Discussion

The present analysis explored Ukrainian refugee women’s beliefs, attitudes, and opinions towards the Romanian and Ukrainian healthcare systems in a comparison model. It also focused on the HPV immunization rates and factors influencing the uptake for themselves and their children. There are limited data available regarding the intricate relationship of the Ukrainian refugees with the Romanian healthcare system [16,33], and no statistics have been published concerning the primary prophylaxis of CC or other non-communicable diseases. As previously highlighted, Ukrainian refugees in Romania face significant social challenges [34], of which healthcare, education, living arrangements, and working opportunities pose noteworthy provocations. Such investigations are paramount, especially since Romania is in a rather nascent state of implementing scaled immunization programs. Therefore, accounting for vulnerable populations and integrating them into national vaccination programs is of vital importance and must be performed early on to ensure that inclusive tactics reach the desired populations and contribute to favorable public health outcomes.

Dumitrache et al. [16] conducted extensive qualitative research on over 120 refugees and asylum seekers in Romania, of which about half were Ukrainians. Cultural, financial, and linguistic issues were notable barriers to accessing the healthcare system in Romania. Our investigation included 105 Ukrainian refugee women while successfully identifying key components that regulate these vulnerable groups’ interactions with the healthcare system and the status of the primary prevention of CC through HPV vaccination. Almost 50 respondents remarked on the contrasting healthcare systems of Ukraine and Romania. While most pinpointed Romanian healthcare workers’ attitudes as better, some impediments occurred in accessing the system. The primary hindrances in accessing healthcare services or immunization programs were the language barrier, the financial burden, and the lack of information.

A previous analysis investigated the addressability of Ukrainian refugees to contingency services in a single center in Romania. With considerable presentations in the emergency department [33], designated programs should be conceived to mitigate the strain on the healthcare system. In a similar manner to urgent care, the non-communicable disease challenges posed by the considerable influx of refugees and asylum seekers in Romania due to the current conflict over the border should be estimated accordingly. Resolutions must be deployed in time, posing preventive care as one of the most important components for a public health system integrating refugees. Of the 105 respondents, only 4 (less than 4%) were vaccinated against HPV, and none remembered what vaccine was used. As formerly uncovered, only a handful of countries from Eastern Europe implemented national HPV vaccination programs [35], and none reached the WHO target [2] of 90% of 15-year-old girls being fully vaccinated against HPV. At the European level, there have been significant discrepancies uncovered regarding the completion of the HPV immunization process for girls aged 15 [36]. Several countries, including Austria, Belgium, Croatia, Cyprus, Czechia, Denmark, Finland, France, Germany, Greece, Hungary, Iceland, Ireland, Italy, Latvia, Lithuania, Luxembourg, Malta, Netherlands, Norway, Poland, Portugal, Romania, Slovak Republic, Slovenia, Spain, and Sweden have implemented gender-neutral programs [37]. Even so, countries like Bulgaria reported rates as low as 9% for completion in this category [36]. Countries like Belgium, Cyprus, Denmark, Finland, Hungary, Iceland, Ireland, Lithuania, Malta, Netherlands, Norway, Portugal, Spain, and Sweden have surpassed the European average of completion of HPV immunization of 63.6% [36]. No data were reported for the Romanian population.

Even if immunization programs are on the rise [38], concerns emerge related to coverage rates, while the Ukrainian region makes no exception. Reported HPV vaccination rates were as low as between 0 and 5.56% in previous investigations [39], in line with the present results and other findings from Ukraine. Similar rates were reported even among well-educated, young, and knowledgeable respondents [40]. Moreover, other concerning analyses uncovered HPV vaccination rates as low as 1.7% among Ukrainian female medical students [41]. Some repeated measurements showed improvements over time, with vaccination rates soaring to just over 6% among respondents [42]. Nevertheless, percentages persist as low as most other Eastern European countries with no HPV vaccination programs implemented, while CCS rates remain unremarkable [43,44]. Vaccine hesitancy among refugees was previously documented [24]. Regardless of the country of origin, most refugees will not complete their vaccination schemes [14]. Therefore, special attention and dedicated efforts must be undertaken to ensure inclusive tactics reach out to these underserved populations, primarily when coming from regions with already subpar coverage.

Low knowledge or health literacy, insufficient information, perceived low susceptibility to infection, and other factors were formerly uncovered as barriers to accessing HPV immunization programs in European women and adolescents [45]. However, most developed countries already have national immunization programs [46]. In a previous comprehensive systematic review, language barriers hindering communication, cultural factors, socioeconomic status, and health literacy were among determinants impeding HPV vaccination in refugee populations [47]. Other investigations reinforced the role of knowledge in HPV immunization for such populations, where rates were uncovered as low as 0.7% for vaccination [48]. Smaller qualitative data suggested that perceived low susceptibility and low knowledge were hindering women refugees from pursuing HPV vaccination [49]. Minimal data were available regarding Ukrainian refugees and their attitudes towards CC primary prophylaxis. However, vaccine hesitancy was regarded as prominent, while HPV vaccination was among the most refused vaccines in a cohort of refugees with children under 18 years of age who were proposed free immunization [50]. This strengthened the foregoing discovery of low uptake within such populations while potentially raising the issue that free vaccination will not suffice to mitigate the issue. As the preceding findings imply, Ukrainian refugees distrusted their vaccination policies and health workers but frequently returned to their home country to access healthcare services [51]. None of the respondents had heard of the HPV vaccine. Although parents reported vaccinating children according to local schedules, they did not complete the schemes themselves [51], similar to our analysis. Interestingly, like our findings, a substantial number of respondents perceived the local health system as better while believing the vaccines were also of better quality or safer. Health system barriers to mandatory or optional children’s vaccinations were also uncovered, such as the lack of interpreting systems, inadequate information, language barriers, and costs, especially regarding HPV vaccination [52]. It is paramount to identify these at-risk groups and conceive tailored vaccination campaigns properly. The present results also uncovered that income and employment in Romania did not interact with the willingness of mothers to vaccinate their children, as opposed to the income and employment rates back in their country of origin. Findings suggest that the refugee status itself is of less importance than the socioeconomic background from which a refugee originates.

## 5. Limitations

Some limitations arise from the present investigation. The invitation system used a snowball sampling method, which is a non-probability technique to recruit participants. This means that a handful of women were recruited directly by their GP (T.A.-C.) or their health mediator. These initial participants recruited the rest of the study population through verbal invitations or social media and group distribution of the questionnaire, or through acquaintances. The use of this so-called chain-referral system inherently has some disadvantages. This method is time-consuming, proven by the long recruitment time of 1 year for reaching 105 respondents. Secondly, this obtained a non-random sample that is difficult to control since the investigators essentially have no power over the ongoing invitations. Lastly, some bias might also be introduced since participants tend to recruit acquaintances like themselves. Nevertheless, this method was among the most suitable techniques for reaching hard-to-access populations such as refugees. There are no readily available lists or sampling frames for these vulnerable populations, and Ukrainian refugee women would not have been accessed as efficiently through other sampling methods. By actively engaging members of the community for recruitment, reluctant participants might feel more confident in answering certain questions, while harder-to-reach populations are engaged. Even so, the method does not enable investigators to accurately estimate the number of invited participants and therefore to provide a percentage of women who agreed to respond.

Another limitation of the analysis is represented by the relatively small sample of respondents, 105 refugee women. Moreover, only four participants were vaccinated against HPV, potentially marking the statistical inferences biased due to the small number of observed events in an already small sample. Several analyses required further grouping of variables to ensure an adequate number of events. This could further introduce bias. Nevertheless, it is paramount to ascertain that the study population consists of a very hard-to-reach vulnerable sample of forcibly displaced people in conditions of conflict in their country of origin. Therefore, even if possible, due to such massive population movements, including a larger sample would require significantly longer times, especially when accounting for the sampling method.

It is vital to address that the current analysis is based in the Cluj-Napoca city in Romania. As previously mentioned, although Romania has a national immunization program and a screening initiative for CC, it lacks an organized program, invitation systems, or national-based registries. Few statistics are available regarding the rates of vaccination and screening addressability, but nevertheless, they remain unremarkable and well below European averages. Although it might seem counterintuitive to assess refugees’ beliefs and attitudes towards HPV vaccination when based in a country that ranks among the first in incidence and prevalence of CC in Europe, it is of vital importance to mention a few aspects. Firstly, the region Cluj-Napoca is among the few in Romania where regional cancer registries are available and considerable efforts are undertaken to design, implement, and sustain a regional-based screening program for CC. It is one of the most developed regions in Romania, and with the presence of a tertiary cancer center in the county that directly contributes to screening and cancer registries, public health policies for non-communicable diseases, and various screening campaigns for underserved populations, it was perhaps the most suitable place to study these aspects in vulnerable populations. Secondly, it is important to evaluate at-risk populations and their hindrances in accessing the healthcare system. While Romania is in a nascent state of developing nationally based immunization campaigns and registries, these vulnerable populations must be considered when developing health policies to ensure equitable health for all.

## 6. Future Directions

Foregoing initiatives already designed and implemented a humanitarian cancer care program for Ukrainian refugees in Moldova and Romania [53]. This proves that public health policies in Eastern Europe could anticipate and mitigate the strain on public health systems that arise when accommodating ethnically diverse populations, especially during times of conflict. With preventive care in mind, such actions could be translated and modified into a tailored prophylaxis program for various communicable and non-communicable diseases addressed to refugees, aiming to increase the uptake of both vaccination and screening. Dedicated personnel with readily available translation experts would mitigate the language barrier, while health mediators could reach these communities.

It is paramount to register most refugees with local GPs who can coordinate the efforts for such programs and oversee their progress regarding the completion of immunization. Actions have been deployed towards implementing screening within family medicine services in Ukraine, and the GPs act as stakeholders. They can even actively participate in procedures, proving their significant part [54]. Results highlighted a higher chance of vaccination for women who had previously engaged in screening practices. This hints at the interplay between primary and secondary prophylaxis, while suggesting that medical specialists should prompt women undergoing one procedure such as screening to also vaccinate. Perhaps designated women’s health clinics for refugees or vulnerable populations should undertake the role of performing both.

Digital storytelling was scoped as a potential culturally adaptable tool in aiding health outcomes [55]. It was previously used as an intervention for mental health and psychological support in forcibly displaced persons throughout Europe [56]. Results also suggest that a significant proportion of respondents were influenced to pursue vaccination after receiving recommendations from non-medical sources, potentially reinforcing the role of alternative strategies.

Mass movements of populations due to the current conflict will result in more ethnically diverse populations across various European countries, raising concerns about proper vaccination coverage and screening practices. Consortia have been assembled to investigate these implications, and it was generally accepted that these forcibly displaced populations should be integrated into the local national vaccination offers [57]. The problem of significant variations concerning optional immunization programs such as HPV throughout European regions remains. Coverages will vary greatly, and estimations have become more difficult in the era of immigration. Catch-up strategies must be deployed; nevertheless, foregoing investigations proposed solutions such as the aid of electronic health records to estimate the extent needed for such strategies [58], as addressability remains low [49]. As Romania has just recently started a National Plan for Combating Cancer that strives to improve screening practices for major oncological pathologies, it is of utmost importance that policymakers are aware of ethnically diverse populations in light of the conflict abroad. They must include vulnerable refugees in the National Immunization Schemes, perhaps using alternative methods for catch-ups. This could be a gateway for creating electronic health records in aiding structured preventive campaigns.

## 7. Conclusions

The present analysis explored Ukrainian refugee women’s beliefs, attitudes, and opinions towards the Romanian and Ukrainian healthcare systems in a comparison model. It also focused on HPV immunization rates, attitudes, and factors influencing the uptake for themselves and their children. It uncovered several key components that could influence the addressability of these at-risk populations. The primary hindrances in accessing health services or immunization programs were language barriers, the financial burden, and a lack of information. General distrust towards the health systems and healthcare workforces was a recurrent theme. Even if a significant portion of respondents were pleased with the Romanian healthcare system, they often traveled back to their home country for medical services. Very low HPV vaccination coverage rates were highlighted along with some variables influencing them, such as relationship status, living arrangements, and previous engagement in screening practices. Perceiving the health officials as proactive regarding optional vaccination programs, such as HPV immunization, or actively receiving recommendations, drove respondents to pursue vaccination. In the context of the current conflict, large numbers of people, including refugees, make up a significant portion of ethnically diverse populations throughout Europe. This raises concerns about vaccination schedules, screening practices, and access to healthcare systems. The challenges highlighted by present findings serve as a foundation for designing future, tailored interventions to improve vaccination rates and complete preventive measures within these populations. As proven by results, a medical specialist’s prompt drove participants to pursue immunization and more than 40% of women received the recommendation from their family doctor in Romania. It is vital to acknowledge the pivotal role of GPs in the primary prophylaxis of CC. Refugees should be enrolled with GPs who can oversee their vaccination schedule (for HPV and perhaps other vaccines) and check for completion rates. As Romania has just recently started a National Plan for Combating Cancer that strives to improve screening practices for major oncological pathologies, it is of utmost importance that policymakers are aware of ethnically diverse populations in light of the conflict abroad. They must include vulnerable refugees in the National Immunization Schemes, perhaps using alternative methods for catch-ups. This could be a gateway for creating electronic health records in aiding structured preventive campaigns. It is essential to ensure inclusive and equitable healthcare services for all to benefit from positive public health outcomes.

## Figures and Tables

**Table 1:** Demographic characteristics regarding continuous data.

Variable	Median	Mean	Std. Deviation	Range	Minimum	Maximum
Age (years)	51	50.01	14.10	56	21	77
ISCED education level	6	5.10	1.67	7	1	8
How long have you been staying in Romania? (months)	12	11.39	6.09	24	0	24
How many times a year do you travel to Ukraine?	0	1.44	2.99	24	0	24

**Table 2:** Demographic characteristics regarding categorical data.

Living Arrangements in Romania	Frequency/105	Percent %
Acquaintance	2	1.90
Alone	25	23.81
Children	24	22.85
Children and husband	16	15.23
Children and relatives	9	8.57
Husband/spouse	16	15.23
Relatives	13	12.38
**Relationship Status**		
Divorced	5	4.76
Married	58	55.23
Not married	21	20
Single	7	6.66
Widowed	14	13.33
**Religious Beliefs**		
Christian	95	90.47
Non-religious	10	9.52
**Employment in Romania**		
No employment	67	63.81
Retired	10	9.524
Yes	28	26.66
**Knowledge of the Romanian language? (self-assessed)**		
Very good	1	0.95
Good	8	7.61
Poor	60	57.14
Very poor	36	34.28

**Table 3:** Gynecological antecedents and characteristics of the respondents.

Last Gynecological Check-Up:	Frequency/105	Percent %
1–3 years	32	40
3–5 years	12	11.42
Less than 1 year	20	19.04
More than 5 years	30	28.57
Never	1	0.95
**Did you ever have a Pap smear or an HPV test?**		
No	59	56.19
Yes	46	43.81
**Did you ever refuse a vaccine?**		
No	94	89.52
Yes	11	10.47
**Did you ever get any HPV vaccination recommendations?**		
No	64	60.95
Yes	41	39.04
**If yes, by whom?**		**Percentage (%) out of 41**
Doctor in Romania	11	26.82
Does not remember	5	12.19
Family doctor in Romania	16	39.02
Family doctor in Ukraine	2	4.87
Friends/family	4	9.75
Internet	2	4.87
Social media doctor	1	2.43
**Are you vaccinated against HPV?**		
No	101	96.19
Yes	4	3.81

**Table 4:** Factors influencing vaccination rates among Ukrainian refugee women.

Variable	Statistics	*p*-Value
Relationship status	Χ^2^ (continuity correction) = 8.78	<0.05
Living arrangements	Χ^2^ (continuity correction) = 9.29	<0.05
Previous engagement in CC screening	OR = 12	0.03
Non-medical other vaccine recommendation	Χ^2^ (continuity correction) = 6.38	0.01
Non-medical HPV vaccination recommendation	Χ^2^ (continuity correction) = 8.31	<0.05
No prior vaccination refusal	Fisher’s exact test = 0.031	<0.05

**Table 5:** Characteristics driving unvaccinated refugee women to pursue HPV immunization.

Variable	Statistics	*p*-Value
Living with family	Χ^2^ = 4.12	0.04
Positive attitudes toward childhood HPV immunization	Χ^2^ = 7.58	0.02
Frequent use of Ukrainian medical services	OR = 4.27	0.02
Native language information could increase vaccination rates	Χ^2^ = 9.53	<0.05
Perceiving Ukrainian authorities as proactive	OR = 3.23	0.02
Perceiving the Romanian authorities as better	OR = 4.72	<0.05

**Table 6:** Characteristics influencing women’s intention to vaccinate their children against HPV.

Variable	Statistics	*p*-Value
Income	Χ^2^ = 10.09	0.01
Employment status	Χ^2^ = 12.97	<0.05
Number of children	Mann–Whitney = 623.5	0.01
Receiving an immunization recommendation	Χ^2^ = 4.00	0.04
Vaccination recommendation from medical sources	OR = 17.27	0.04
Willingness to pursue own HPV vaccination	OR = 3.41	0.01
Not remembering children’s vaccination/refusing vaccination	OR = 0.29	<0.05
Healthcare system access after moving to Romania	OR = 11.71	<0.05
Perceiving Romanian authorities as encouraging	OR = 3.78	<0.05
Willingness to seek medical care after moving to Romania	OR = 5.09	<0.05
Sufficient information is available in native language	OR = 0.192	0.02
Ukrainians have safety concerns regarding the HPV vaccine	Χ^2^ = 7.21	0.02
Positive attitude regarding the HPV vaccine	OR = 3.33	<0.05

## Data Availability

Data available on request.

## Supplementary Materials

The following supporting information can be downloaded at: https://www.mdpi.com/article/10.3390/healthcare13141744/s1, S1: Questionnaire.
