# Supplementary material for: Exploring Ukrainian Refugee Women’s Beliefs and Concerns About Healthcare Systems, with a Focus on HPV Immunization Practices: A Mixed-Methods Study on Forcibly Displaced Populations in Romania
Source: Healthcare (Basel). 2025 Jul 18;13(14):1744. doi: 10.3390/healthcare13141744 (PMC12294297; doi:10.3390/healthcare13141744)
Supplement: Supplementary file 1 [file healthcare-13-01744-s001.zip › healthcare-3724391-supplementary.pdf]

I. Demographic Questions

1. Age (years)
2. Education
3. ISCED Education Level
4. Relationship status   Religious beliefs
5. Who are you living with in Romania?
6. Who did you live with before moving to Romania?
7. Income in Ukraine
8. Income in Romania
9. Ukrainian employment
10. Employment in Romania
11. How long have you been staying in Romania? (months)
12. How many times a year do you travel to Ukraine?
13. How do you describe your knowledge of the Romanian language?

a) very good

b) good

c) poor

d) very poor

14. Last gynaecological check-up:

a) less than a year ago

b) 1 – 3 years ago

c) 3 – 5 years ago

d) more than 5 years ago

15. Did you ever have a Pap smear or an HPV test?

If yes, did you ever have some abnormalities on the Pap smear or HPV test?

16. Your vaccination schedule - Do you remember what you were vaccinated for to date (mandatory or non-mandatory vaccines)?
    Please, specify:
17. Did you ever get any vaccination recommendations?
    If yes, by who?
    If yes, for what vaccine?
18. Did you ever refuse a vaccine?
    If yes, please specify:
19. Did you ever get any HPV vaccination recommendations?

If yes, by who?

If yes, for what vaccine?

20. Are you vaccinated against HPV?
    If yes, what vaccine and how many doses?
    If not, would you like to get the HPV vaccine? Why?
21. Do you have children?
    If yes, how many?
    If yes, how many of them are boys, and how many are girls?
    If yes, do you remember what your child was vaccinated for? Please, specify:
    If yes, do you remember any vaccination refusals for your children? Please, specify which vaccine and the reason for rejection:
    Would you like to vaccinate your children against HPV? Why?2

II. Topic questions

ACCESS TO HEALTHCARE SERVICES IN UKRAINE AND ROMANIA

1. Did your access to healthcare change after moving to Romania? (Yes/No)
    a) Please specify the reasons:

b) Please specify if there are any differences between Ukrainian and Romanian healthcare services
c) Please specify if there are any similarities between Ukrainian and Romanian healthcare services
d) Please specify what would help to improve your access to healthcare services in Romania

2. Did your willingness to seek medical care change after moving to Romania? (Yes/No)
   a) Please specify the reasons:
   b) Please specify what would help to improve your willingness to seek medical care in Romania
3. Did you use state-offered services or prefer the private sector in Ukraine?
   a) Why?
4. Do you use state-offered services or prefer the private sector in Romania?
   a) Why?

5. Did you have access to primary prevention in Ukraine?

6. Did you have access to primary prevention in Romania?

7. Did you notice any differences between Ukrainian primary prevention and Romanian? (including vaccination) (Yes / No)

a) Please specify:

INFORMATION REGARDING THE HPV VACCINE

8. Do you think sufficient information is available in the Ukrainian language about HPV vaccinations in Romania? (Yes / No)

9. What were your sources of information about the HPV vaccine in Ukraine?

10. What are your sources of information about the HPV vaccine in Romania?

HEALTHCARE ACCESS AND DELIVERY OF THE HPV VACCINE

11. Do you use medical/dental services in Ukraine when you travel there on holiday? (Yes/No)

a. What services do you use?

b. Why?

12. How do you feel using Ukrainian medical services compared to Romanian ones?"

13. Do you prefer to use Ukrainian medical services or Romanian ones?

a. Why?

14. What do you think is the attitude of Ukrainian authorities regarding vaccination?

a. Why?

15. What do you think is the attitude of Ukrainian authorities regarding HPV vaccination?

a. Why?

16. What do you think is the attitude of Romanian authorities regarding vaccination?

a. Why?

17. What do you think is the attitude of Romanian authorities regarding HPV vaccination?

a. Why?

18. Are there enough vaccines for everyone in need in Ukraine?

19. What factors could help increase the HPV vaccination in Ukraine?

a. Why?

20. What do you think about the access to the HPV vaccine in Romania?

21.What factors could help increase HPV vaccination for Ukrainians in Romania?

a. Why?

22.Do you think free HPV vaccination could increase addressability?

TRUST IN HEALTHCARE SERVICES

23. Do you think Ukrainians in Romania have confidence in Romanian healthcare workers? (Yes/No)

a. Why?

b. Who? (doctors, nurses, midwives, other employees)

24. Do you think Ukrainians in Romania have confidence in Ukrainian healthcare workers? (Yes / No)

a. Why?4

b. Who? (doctors, nurses, midwives, other employees)

CONFIDENCE IN VACCINATION POLICIES AND DELIVERY

25. Are you aware that vaccination schedules are slightly different in Romania and Ukraine? (Yes/No)

26. How do Ukrainians perceive the Romanian vaccination program in general?

27. What are the differences in your perception between Romanian and Ukrainian vaccination systems?

28. Have you experienced the Romanian vaccination system? (Yes / No)

a. How?

29. Can one get a false vaccination certificate in Ukraine?

How about in Romania?

CONFIDENCE IN THE VACCINES PROVIDED (SAFETY/ QUALITY/ IMPORTANCE OF VACCINATION)

30. What do you think is the attitude of Ukrainians regarding HPV vaccination?

31. What is your attitude regarding HPV vaccination?

32. What do you think are the reasons for HPV vaccination refusal?

33. What do you think are the reasons for HPV vaccination acceptance?

34. Do you think that Ukrainians consider HPV vaccination safe? (Yes / No)

35. Do you think that Ukrainians consider HPV vaccination effective? (Yes / No)

How important is it that (1 - very unimportant, 2 - unimportant, 3 - neutral, 4 - important, 5 - very important)

a. children receive routine childhood immunizations (such as measles, mumps, rubella and others)

b. seasonal influenza vaccine is administered in school children/older people/ pregnant women

c. teenage girls are vaccinated against HPV

d. vaccinations are current in pregnant women (smallpox/rubella/warts)
